# Brain tissue oxygen reactivity: clinical implications and pathophysiology

**DOI:** 10.3389/fphar.2014.00100

**Published:** 2014-05-20

**Authors:** Gurgen Harutyunyan, Harutyun Mangoyan, Gagik Mkhoyan

**Affiliations:** ^1^Department of Emergency Medicine, Hospital 9 De OctubreValencia, Spain; ^2^Department of Intensive Care, Erebuni Medical CenterYerevan, Armenia

**Keywords:** brain tissue oxygen reactivity, cerebral blood flow, blood buffers, brain metabolism, cerebral metabolism

## Introduction

It is generally accepted that PbO_2_ reflects the balance between O_2_ delivery and consumption (Diringer et al., 2007; Diringer, 2008). However, implementation in the perioperative period of various ventilatory modes using high FiO_2_ leads to a dramatic and non-physiologic increase in PbO_2_ with approximating levels of 147 ± 36 mmHg (McLeod et al., 2003). This phenomenon doesn't correlate with the extent of slight increase in arterial O_2_ content. At the same time, the jugular venous PO_2_ increases only slightly (37–40 mmHg) (Forkner et al., 2007). Moreover, hyperoxia does not affect significantly the regional CBF, and there is no improvement in cerebral metabolism with oxygen therapy (Magnoni et al., 2003; Diringer et al., 2007; Diringer, 2008; Xu et al., 2012).

The PbO_2_ increase is more pronounced in edematous (but not necrotized) brain tissues compared to normal areas (Meixensberger et al., 1993). Although, this can be considered a positive phenomenon, it masks the real state of rCBF and local oxidative metabolism. Recording of high PbO_2_ absolute values may create a false impression of safety and negatively impact the clinical decision making. Apparently, better indicators of the status of energy exchange in the brain tissue are needed for practical use in the perioperative and critical care settings.

## Brain tissue oxygen reactivity: clinical implications

Dynamic assessment of relative changes in brain oxygenation to monitor the brain functionality is a better approach compared to relying on a single parameter. With such monitoring, both the current status of brain tissue oxygenation and the functional reserve capabilities can be accomplished.

Brain tissue oxygen reactivity (BTOR) is the measure (in percents) of PbO_2_ changes relative to changes in PaO_2_ (ΔPbO_2_/ΔPaO_2_) with oxygen inhalation (Johnston et al., 2003). The latter parameter can be easily adjusted to reach BTOR optimal values. The technique of measurement includes increasing the FiO_2_ up to 1.0 with simultaneous recording of the PaO_2_ and PbO_2_ values.

Literature reports indicate that high BTOR values within the first 24 h after TBI are considered an indicator of unfavorable outcome and negatively correlate with the Glasgow Outcome Score (van Santbrink et al., 1996; Menzel et al., 1999).

It is not mandatory to apply the maximal FiO_2_ of 1.0 to calculate the BTOR. Any other high inspired O_2_ levels can be applied that will produce significant PbO_2_ changes within 20 min. Such a time period is considered the minimal required interval adequate for equilibration and meaningful assessment. During this short period, the respiration, regional metabolism and the rCBF are assumed to remain stable, and the calculated values of ΔPbO_2_/ΔPaO_2_ will indirectly characterize the rCBF.

Low BTOR is considered a positive phenomenon even when the absolute PbO_2_ values decrease, unless regional hypoperfusion (<20 ml/100 g/min) exists (Hlatky et al., 2008). Simultaneous elevations of PbO_2_ and ΔPbO_2_/ΔPaO_2_ values reflect the imbalance between the oxygen delivery and consumption.

Under normal cardio-respiratory conditions, when the right to left pulmonary shunting is negligible, the FiO_2_ is proportional to PaO_2_. On the other hand, PbO_2_ itself correlates with PaO_2_. Therefore, one can presume that FiO_2_ is proportional to PbO_2_. Taking this into account, the formula used to calculate the BTOR can be modified to evaluate the correlation between the changes in PbO_2_ and FiO_2_. This new parameter (ΔPbO_2_/ΔFiO_2_) is considered an equivalent of BTOR and can be easily calculated. This is a simple and practical approach to BTOR assessment that can be readily used at bedside. Such an approach will allow for dynamic assessment of tissue oxygen reactivity.

## BTOR: pathophysiology

In order to illustrate the importance of BTOR as an ultimate indicator of balance between the rCBF, oxygen delivery and consumption and justify the need for its monitoring, the hypothesis of hyperreactive, non-physiologic, luxurious PbO_2_ elevation is proposed.

We hypothesize that the significant increase of PbO_2_ with hyperoxia in the injured brain is explained by an excessive right shift of the oxyhemoglobin dissociation curve with resultant significant reduction in hemoglobin's affinity to oxygen molecules at the microcirculatory level. This is a result of a mismatch between the rCBF and existing cerebral metabolic rate of oxygen (CMRO_2_), which leads to accumulation of CO_2_, converted by erythrocyte carboanhydrase into HCO^−^_3_ and H^+^ ions (at a 1000 times faster rate compared to plasma and extracellular space). It is known that CO_2_ and H^+^, which are produced during the tissue metabolism, are heterotropic effectors of hemoglobin that enhance oxygen release (Berg et al., 2002). The latter ions bind to hemoglobin with release of oxygen. With decrease in rCBF and/or relative increase of CMRO_2_, hemoglobin gets saturated with protons and practically loses its affinity to oxygen in the microcirculatory bed.

The role of CO_2_-induced local increase of PO_2_ is particularly important in the brain tissue where, under normal conditions (glucose-dependant metabolism without chronic fasting), the respiratory quotient equals 1 and the CO_2_ production almost 1.25 times exceeds that of the other tissues.

According to the above mentioned considerations, the rCBF determines PbO_2_ values via two principal mechanisms: (a) as an oxygen delivery mechanism within the arterial compartment and (b) via a “non-physiologic” right shift of the oxyhemoglobin dissociation curve as a result of decreased removal rate of the flow-dependent metabolites in the microcirculatory bed.

Many drugs and techniques used commonly during therapy of severe TBI, including manitol, sodium thiopenthal, ketorolac, nimodipine, intra-arterial papaverine, hypothermia, deep sedation, etc., can reduce the PbO_2_ in the damaged tissue (Steiner et al., 2001; Gupta et al., 2002; Stiefel et al., 2004, 2006; Sakowitz et al., 2007). On the other hand, the effects of medically induced augmentation of cerebral perfusion pressure on cerebral oxygenation are difficult to predict (Sahuquillo et al., 2000; Imberti et al., 2002; Le Roux and Oddo, 2013). In addition, Zygun et al. (2009) showed that even though transfusion of packed red blood cells in TBI patients may improve the brain tissue oxygenation, it won't have an appreciable effect on cerebral metabolism (Zygun et al., 2009). Thus, there is a complex interaction of multiple factors influencing the functional and metabolic activity of the injured brain including injury-related pathological mechanisms, drugs and methods used to manage these patients. Their overall effects are not straightforward and cannot be anticipated easily in an individual case. Apparently, Monitoring of PbO_2_ in these patients will not provide reliable feedback and may be misleading in some cases. It is not justified to treat the severe TBI patients relying only on the PbO_2_ as an indicator of adequacy of cerebral metabolism. Instead, dynamic oxygen reactivity should be routinely monitored as an indicator of overall brain tissue oxygenation and metabolism.

## Calculations

Assuming CBF and CMRO_2_ stability during oxygen therapy and equivalence of PbO_2_ with capillary PO_2_, (Kett-White et al., 2002) we can modify the standard formula for calculation of arterio-venous difference in oxygen (Kett-White et al., 2002) to determine the changes in hemoglobin saturation in the capillary blood:

$$\begin{array}{l} \text{Sv}._{\text{a}}{\text{O}}_{2}-\text{Sv}._{\text{b}}{\text{O}}_{2}=[{\text{Ct}}_{\text{a}}{\text{O}}_{2}\left(\text{a}\right)-{\text{Ct}}_{\text{b}}{\text{O}}_{2}\left(\text{a}\right)\\ \;-0.003*\left({\text{Pb}}_{\text{a}}{\text{O}}_{2}-{\text{Pb}}_{\text{b}}{\text{O}}_{2}\right)]/1.34\text{xHb} \end{array}$$

where Sv._a_O_2_ and Sv._b_O_2_ are oxygen saturation at distal microcirculatory level after and before inhalation of oxygen; Ct_a_O_2_ (a) and Ct_b_O_2_ (a) are arterial oxygen content values after and before initiating oxygen therapy; Pb_a_O_2_ and Pb_b_O_2_ are PbO_2_ values after and before starting inhalation of oxygen; and Hb is hemoglobin concentration in g/dL.

For example, if we increase PbO_2_ from P_50_ = 35 mmHg (if hemoglobin saturation is 0.5 or 50%) to 100 mmHg and assume a change in CtO_2_ (a) equal to 1 vol. %, the hemoglobin saturation at distal microcirculatory level will change in the following way (assuming a hemoglobin concentration 12 g/dL):

$$\begin{array}{l} {\text{Sv}}_{\text{a}}{\text{O}}_{2}-{\text{Sv}}_{\text{b}}{\text{O}}_{2}=\left[1-0.003\;*\;\left(100-35\right)\right]\\ \;/1.34\text{x}12=0.05\text{ or }5\;\% \end{array}$$

This means that the distal microcirculatory oxygen saturation under these arterial conditions (PbO_2_ = 100 mmHg) will only increase 50% + 5% = 55%.

Calculations show the weak affinity of hemoglobin to oxygen under these conditions which results in allocation of additional oxygen amounts out of hemoglobin with creation of abnormally high PbO_2_ in injured brain tissue areas.

## Conclusions

Monitoring of BTOR or its equivalent ΔPbO_2_/ΔFiO_2_ is indicated during the intensive therapy of TBI patients. Both indices reflect the actual status of cerebral oxidative metabolism and help to reduce the risk of management errors which are otherwise masked by high FiO_2_-induced “adequate” PbO_2_ absolute values.

Blood transfusions, controlled hyperventilation and restoration of the regional acid-base balance should be performed under the guidance of above mentioned indices.

Further studies will help to establish the role of BTOR and ΔPbO_2_/ΔFiO_2_ monitoring in assessment of metabolic changes and adaptations taking place in the injured brain during the acute phase of TBI.

## Disclosure

The authors did not receive any financial or other support related to this work.

### Conflict of interest statement

The authors declare that the research was conducted in the absence of any commercial or financial relationships that could be construed as a potential conflict of interest.
